# Reasons for not undergoing cervical cancer screening: perspectives from women and health care providers in Addis Ababa: a qualitative study

**DOI:** 10.3389/fonc.2025.1456804

**Published:** 2025-04-02

**Authors:** Ebrahim Mohammed, Mirgissa Kaba, Girma Taye, Mathewos Assefa, Ahmedin Jemal, Adamu Addissie

**Affiliations:** ^1^ Department of Reproductive, Family and Population Health, School of Public Health, Addis Ababa University, Addis Ababa, Ethiopia; ^2^ Department of Preventive Medicine, School of Public Health, Addis Ababa University, Addis Ababa, Ethiopia; ^3^ Department of Epidemiology and Biostatistics, School of Public Health, Addis Ababa University, College of Health Sciences, Addis Ababa, Ethiopia; ^4^ Department of Oncology, College of Health Sciences, School of Medicine, Addis Ababa University, Addis Ababa, Ethiopia; ^5^ Surveillance & Health Equity Science, American Cancer Society, Atlanta, GA, United States

**Keywords:** cervical cancer, cervical cancer screening, perspectives on cervical cancer, women, healthcare providers

## Abstract

**Background:**

Cervical cancer is a major public health problem in low-income countries, including Ethiopia. Various pieces of evidence show that the uptake of cervical cancer screening is low in Ethiopia. The reasons for this low uptake of cervical cancer screening have not been well documented.

**Objective:**

The aim of this study is to explore the reasons for not taking up cervical cancer screening and gather the perspectives of women and healthcare providers in Addis Ababa, Ethiopia.

**Methods:**

Adult women and healthcare providers participated in the study. Eleven focus group discussions were conducted with women from the community. A total of 18 key Informant interviews were conducted with healthcare professionals who providing cervical cancer screening services and family health team leaders. Interviews and discussions were audio recorded, transcribed, and coded. We used MAXQDA software v.20 for data reduction to facilitate thematic analysis and interpretation.

**Results:**

Eleven focus group discussions and 18 key informant interviews were conducted. In this study, individual-level barriers, such as low knowledge of cervical cancer and screening, feeling healthy, fear of the screening procedure and results, fear of not being cured, fear of divorce, stigma and discrimination, preference for female healthcare providers, and spousal disapproval or resistance, were identified as the main reasons for the low uptake of screening. Community-level barriers such as perceiving cervical cancer as a deadly disease; misconceptions, such as screening causing infertility, and the absence of open discussion, were also found to contribute to low screening uptake.

**Conclusion and recommendations:**

Knowledge about cervical cancer and screening was found to be inadequate. Individual and community-level socio-cultural barriers were identified as reasons for the low uptake of screening. Therefore, it is crucial to conduct behavioral change and communication activities at both the individual and community levels to increase knowledge of cervical cancer and screening, reduce sociocultural barriers, and improve the uptake of cervical cancer screening.

## 1 Introduction

Cervical cancer is the fourth most common cancer and is a major public health issue worldwide (1). It is the second most common type of cancer and causes an estimated more than 4,700 deaths annually among women in Ethiopia (2, 3). The incidence rates of cervical cancer have decreased by more than half in many high-income countries over the past five decades, mainly due to widespread accessibility of screening services. However, in low- and middle-income countries, the rates of cervical cancer remain high due to the absence of organized screening services at the community level. As a result, low-income countries contribute over 80% of the global burden of cervical cancer (4, 5). The high burden of the disease in low-income countries could be attributed to the inaccessibility of screening services and a lack of knowledge about the benefits of early screening, detection, and treatment.

The incidence rate of cervical cancer screening in Addis Ababa city is low, ranging from 2.0% to 25.5% (6–8). Most cases of cervical cancer are detected at advanced stages, leading to poor prognosis due to delayed diagnosis (9). This late stage of diagnosis is attributed to a lack of screening services at the community level, low awareness, other socio-cultural barriers such as the influence of spousal support, the distance of health facilities providing screening services, and other organizational barriers related to the screening services.

The World Health Organization (WHO) has introduced a global strategy to accelerate the elimination of cervical cancer by 2030. The strategy aims to achieve 90% vaccination coverage, 70% screening coverage, and 90% treatment coverage in each country (10). For the implementation of “See and Treat” approaches, the WHO recommends visual inspection with acetic acid (VIA) or visual inspection with Lugol’s iodine (VILI) every three years for women aged 30-49 years in low-resource countries (11). Following the WHO strategy, Ethiopia’s 2015 National Cervical and Breast Cancer Prevention and Control guideline was introduced to combat cervical cancer by implementing a national cervical cancer screening program using a “see and treat” approach with visual inspection of the cervix with acetic acid (VIA) (2).

Barriers to the uptake of screening have been widely studied and documented in various parts of the world. In a study conducted in the UK, several barriers were identified, such as reluctance to know the test results, the belief that the test is unnecessary for asymptomatic women, lack of trust in healthcare services, and inconvenient appointment times (12). Similarly, a Canadian study highlighted barriers such as inconvenient clinic hours, procedural obstacles, and long travel distances to screening services (13). In a study in the USA, among Asian-American women, barriers like psychosocial factors, religious beliefs, limited knowledge, and inadequate access to healthcare were identified (14). Another study conducted in the USA, specifically in Minnesota among Somali women in key informant interviews, found that barriers at the individual level (knowledge limitations, religious beliefs, and feelings of pain, fear, and embarrassment) and the community level (culture and modesty, stigma, language problems, and trust in the healthcare system) contribute to low uptake of screening (15).

In one of the focus group discussions in Ghana, it was shown that the uptake of cervical cancer screening was low due to low-level awareness of screening and personal factors, screening procedure, screening facilities, and low income (16). Another study conducted in Kenya indicated that barriers such as spousal approval, stigma, embarrassment, fear of infertility as a result of speculum examination, fear of residual effects of test results, lack of awareness and knowledge, and religious or cultural beliefs were identified as barriers (17).

Studies conducted in Ethiopia, including Addis Ababa, have indicated that lack of awareness, negative community perceptions, financial problems, stigma, and long waiting times as barriers to the uptake of cervical cancer screening (18). Another study conducted in Jimma and Addis Ababa (through focus group discussions) showed that lack of awareness, perceived etiology, stigma, and limited access to cervical cancer screening services were also barriers to the uptake of screening (19).

There is a lack of evidence that could show the perspectives of women, cervical cancer screening service providers, and healthcare leaders on cervical cancer and screening in Addis Ababa. Therefore, this study aims to explore the perspectives of women and service providers regarding cervical cancer and screening uptake among women in Addis Ababa by organizing focus group discussions among women and conducting key informant interviews among service providers.

## 2 Materials and methods

### 2.1 Study setting and design

Addis Ababa, the capital city of Ethiopia, is experiencing rapid growth and is home to various African and international diplomatic organizations. The city has a population of over 5.7 million (20) and is administratively divided into 11 sub-cities and 111 districts. Within the city, there are 99 public primary care health centers and 12 public hospitals; all of which offer cervical cancer screening services. To investigate the reasons for the low uptake of cervical cancer screening, an explanatory phenomenological qualitative study was utilized.

### 2.2 Study population and participant selection

The study was conducted among adult women, health care providers (cervical cancer screening service providers), and district family health team leaders in Addis Ababa. Women aged 25–65 living in Addis Ababa were grouped into different categories, such as politically organized groups (Women Development Army and Women League groups) and socially organized groups (community members and leaders like “Idir” local and cultural women organizations). These groups were purposively selected and included in the focus group discussions to ensure diverse viewpoints. Health care workers providing screening service and health center Family Health Team leaders working on cervical cancer preventions and control activities were purposively included in the key informant interviews to understand the thoughts and perspectives of women on screening uptake.

**Figure 1:**
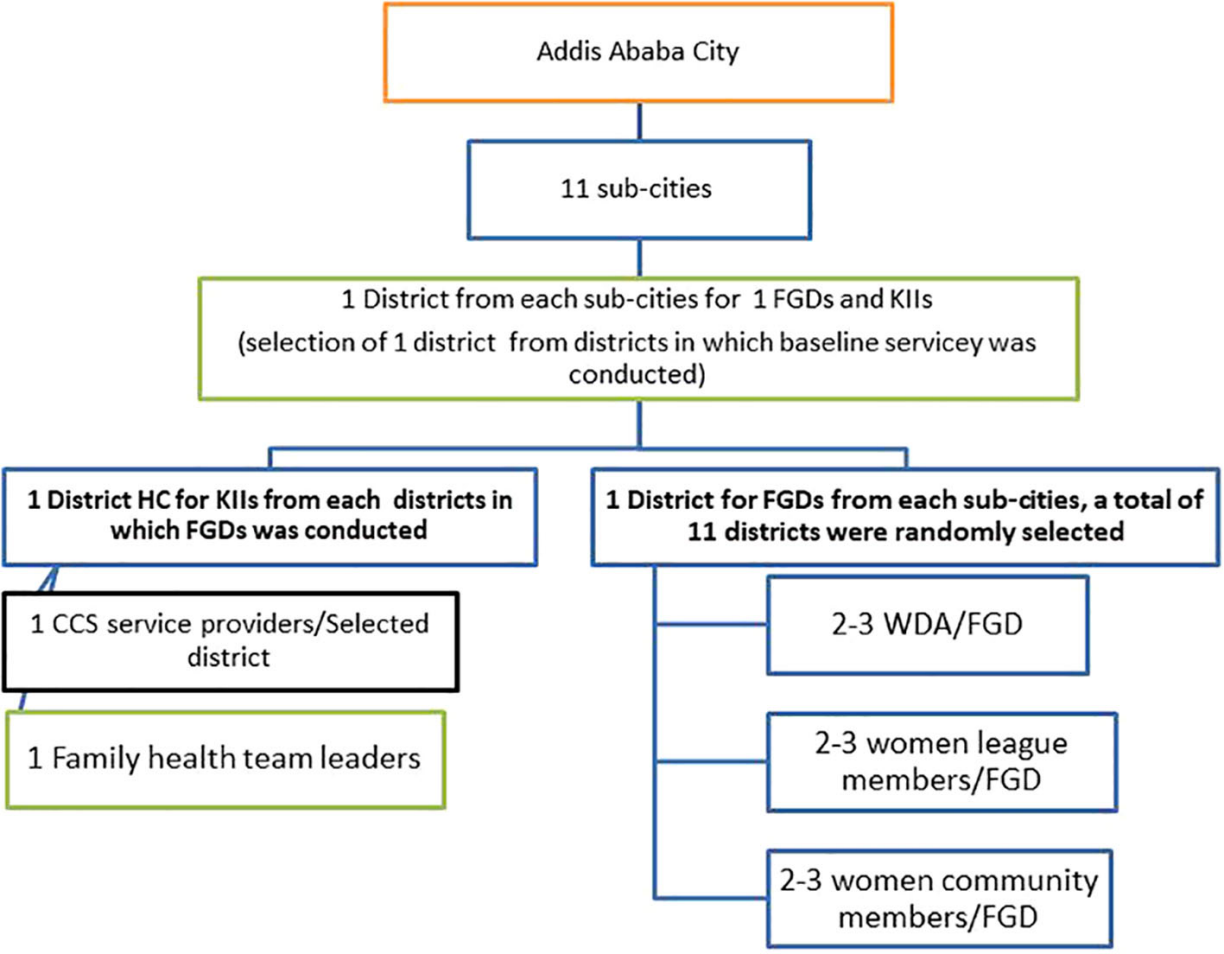
Selection techniques of the study participants from women in Addis Ababa.

One district was randomly selected from each sub-city where the baseline survey was conducted. Six to nine women were included in each focus group discussion, and one screening service provider and one family health team leader were included in key informant interviews from each selected district health center (See **Figure 1**).

### 2.3 Data collection tools and procedure

The data collection guide was developed based on the study objectives for both the focus group discussions (FGD) and key informant interviews. The guide included open-ended questions about socio-demographics, awareness, benefits of screening, knowledge of risk factors for cervical cancer and prevention, reasons for not undertaking screening, and community perceptions and expectations. The focus group discussion guide was pre-tested in Kolfe Keraniyo sub-city, a district not selected for focus group discussion. Data was collected through focus group discussions with 6–9 discussants and key informant interviews in each district. Focus group discussions were held in selected health centers within the districts, in meeting halls or offices, to ensure a noise free environment. Audio recorders were used to capture the voices of discussants and key informants. Two trained data collectors were involved in the data collection. One recorded the audios and the other facilitated the discussion during focus group discussions. The focus group discussions lasted 50 - 70 minutes, depending on the interaction among discussants. Key informant interviews with health care providers were also conducted in their work places and lasted 20-30 minutes. Both focus group discussions and key informant interviews were recorded for later analysis, with field notes taken to aid the data analysis.

### 2.4 Data analysis

The recorded voices were transcribed after multiple listens to the recordings in the local language (Amharic). They were then translated into English by foreign language experts and back-translated into the local language by another expert. MAXQDA software version 20 was used to code the transcripts. Two independent coders created the codebook after carefully reading the transcripts. A discussion was held to identify any differences in the codes, and finally, an agreement was reached on the codes. Then, the verbatim statements were then coded. The principal investigator actively participated in the interviews and listened to the recordings multiple times before transcribing them. Member checking was done with two KII participants to ensure the credibility of the data. A thick description was used to ensure the transferability of the findings. Four thematic categories were identified: 1) Awareness of cervical cancer and screening, 2) Knowledge of cervical cancer and screening, 3) Socio-cultural barriers, and 4) Women’s expectations. Additionally, three to four sub-themes were identified under each theme. Finally, a thematic analysis was conducted.

### 2.5 Ethics approval

Ethical approval was obtained from Addis Ababa University College of Health Sciences Institutional Review Board number 081/22/SPH. Written permission was also obtained from the Addis Ababa City Health Bureau and each sub-city. The objectives of the study were explained to all participants, and informed consent was obtained to ensure voluntary participation. Focus group discussion Participants were reimbursed for transportation expenses after each focus group discussion session.

## 3 Result

### 3.1 Study participants characteristics

Ninety-two (92) participants took part in 11 focus group discussions, with an average of 8 participants, and eighteen (18) health care providers (HCPs) participated in key informant interviews. More than half of the participants of the focus group discussion and key informant interview participants were aged 31 to 40 years. The median age of focus group discussion participants was 37 years (IQR = 12.5), while that of key informant interview participants was 32 years (IQR = 6.25). Half of the focus group discussion participants had a secondary level of education, and more than two-thirds of them were housewives (homestay mothers). Nearly half of the focus group discussion participants belonged to the women’s development army group. The majority of the key informant interview participants were first-degree holders, midwives by profession, and more than half of them were cervical cancer screening service providers (See **Table 1**).

**Table 1:** Socio-demographic characteristics of study participants, Addis Ababa.

Characteristics of FGDs participants	Frequency	%	Characteristics of KIIs participants	Frequency	%
Age category			Age category		
20-30 years	18	19.6	20-30 years	6	33.3
31-40 years	47	51.1	31-40 years	10	55.6
41-50 years	22	23.9	>40 years	2	11.1
>= 51	5	5.4	Educational status		
Educational status			Diploma	1	5.6
No Education	7	7.6	1st Degree	15	83.3
Primary	32	34.8	2nd Degree	2	11.1
Secondary	46	50.0	Profession		
Diploma & Above	7	7.6	Nurse	5	27.8
Occupation			Midwife	8	44.4
Daily Laborer	1	1.1	Public health professionals	4	22.2
House wife	62	67.4	MPH	1	5.6
Govt. Worker	9	9.8	Role in the Health institution		
Private work	20	21.7	Screening service Provider	10	55.6
Role in the community			Family health team leader	6	33.3
Women development army	42	45.7	Health center head	2	11.1
Community member	28	30.4			
Women league	15	16.3			
Others	7	7.6			

### 3.2 Emergent themes

**Figure 2:**
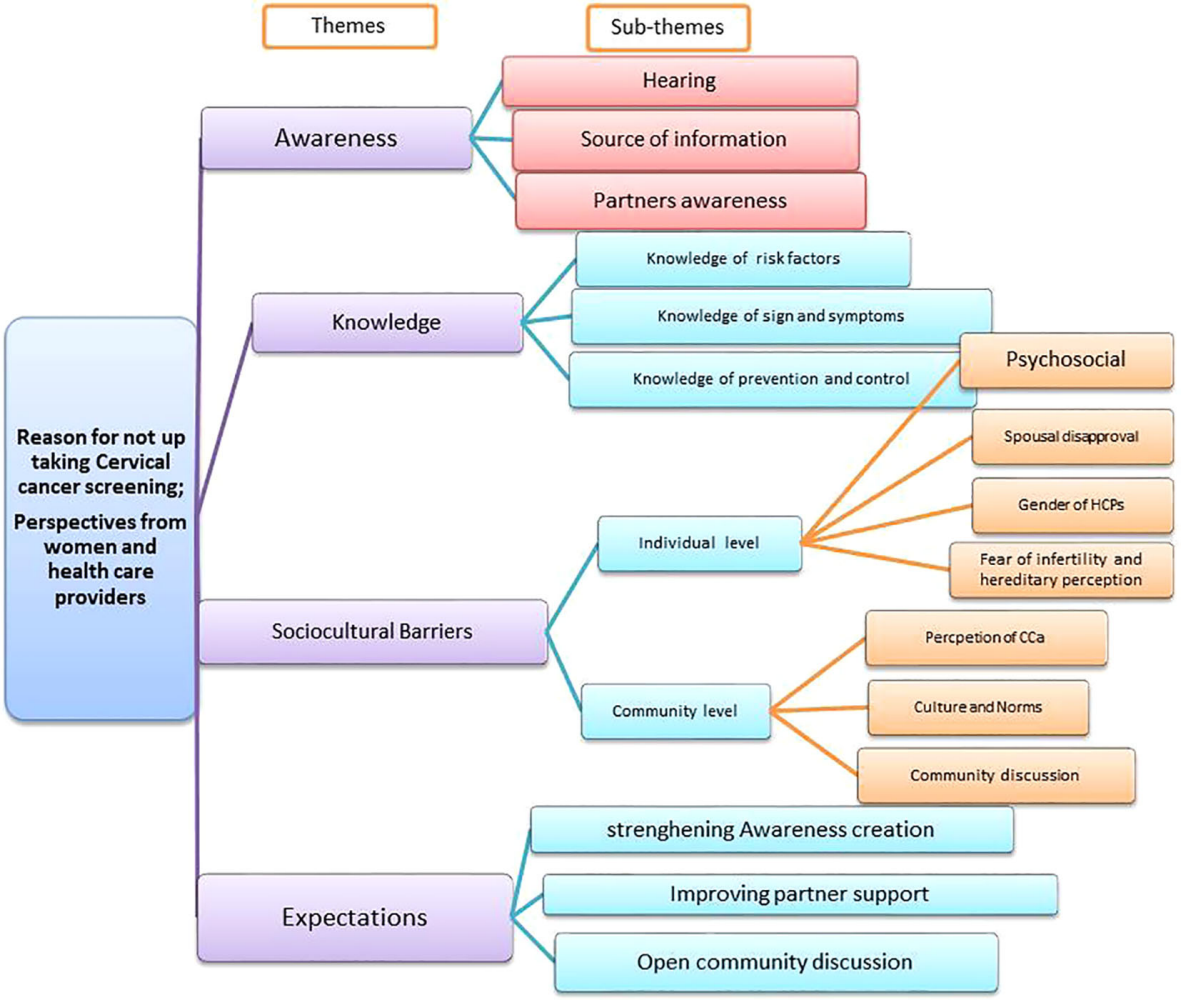
Perspectives on CCa with identified themes and sub-thematic categories, Addis Ababa.

The study was organized around four emerging themes: 1) awareness of cervical cancer and screening; 2) knowledge of cervical cancer risk factors, symptoms, control and prevention; 3) socio-cultural barriers; and 4) women’s expectations. Perspectives of adult women and health professionals on cervical cancer screening were discussed separately within their respective groups. These themes were identified through code analysis (See **Figure 2**).

#### 3.2.1 Theme 1: awareness about cervical cancer and screening

##### 3.2.1.1 Hearing about cervical cancer and screening up take

During the focus group discussion, participants were asked if they or other women in the community were aware of cervical cancer and screening. Similarly, key informant interview participants were asked if the women in their health center`s catchment area had heard about cervical cancer and screening. The majority of focus group discussion and key informant interview participants reported that most of the women were familiar with cervical cancer and screening. Focus group discussion participants said;

*I think most of the women had heard about it. The health extension workers teach us during health home visits and community health meetings. I believe most women had heard of it. (FGD group 2 P3, FGD group 3 P5, FGD group 4P 2, FGD group 6P1, FGD group 6P2)*

*One of the key informants said*

*It is a priority service area for our institution. Our family health team provides health education to women during home visits. I believe most women in our district have heard about cervical cancer and screening. (HCP, M30, family health team leader).*

##### 3.2.1.2 Source of information

Women who participated in the focus group discussion were asked about the source and their choice of information sources. Approximately seventy-five percent of focus group discussion participants reported that they heard from mass media, half reported hearing from healthcare workers, and one-fourth reported hearing from multiple sources such as healthcare providers, health extension workers, and mass media. During the discussion, we also assessed the preferences for information sources. Some participants mentioned that they prefer receiving information from healthcare providers because it allows them to seek clarification for any questions they may have. Focus group participants mentioned as follows:

*Women may hear about cervical cancer and screening from health extension workers, healthcare providers, and TV. However, it would be better if women hear it from healthcare professionals as they can have the time and opportunity to ask questions about cervical cancer and cervical cancer screening that may be unclear to them (FGD group 1 P1, FGD group 2 P3, FGD group 3 P5, FGD group 4P 2, FGD group 6P1, FGD group 6P2 and almost all KIIs Participants).*

*Women can hear information from various sources such as TV, HCPs, and social media. It is better to hear from a health professional like me, and when they have questions, they will get answers (HCP, F31 years old, screening service provider).*

##### 3.2.1.3 Spousal hearing and awareness

Spousal awareness of cervical cancer and cervical cancer screening is important to support women in accessing cervical cancer screening (21). Women in the focus group discussions were asked if their partners or spouses had heard about cervical cancer and cervical cancer screening. Key informant interview participants were also asked if males are aware of cervical cancer and cervical cancer screening. Some of them replied that

*“Most men didn’t have much information about cervical cancer and screening, except for what they may have heard on TV. Health extension workers are unable to teach males during home visits as most men are at work when health extension workers visit homes to educate the community (FGD group1 P3, FGD group1 P5, and FGD group3 P7).”*

*Most men are unaware of cervical cancer. We have only provided education to women during home visits, not to men. However, well-educated men may have read about it and become aware. I believe that the majority of men have not heard of it. (HCP, F31-year-old screening service provider and HCP, F33-year-old family health team leader)*

#### 3.2.2 Theme 2: knowledge related to cervical cancer And screening

##### 3.2.2.1 Knowledge of risk factor

Knowledge of risk factors is an important aspect of cancer prevention and control. Women in the focus group discussion and key informant interview participants were asked whether they knew about cervical cancer risk factors or not. Most of the focus group discussion participants did not know the risk factors, and more than four-fifths of key informant interview participants said that most women did not know the risk factors. The study participants said:

*“I had heard about cervical cancer, but I didn’t know what causes it or what exposes someone to cervical cancer. I don’t think most women are aware of the risk factors of cervical cancer.” (FGD group 2 P6, FGD group 3 P5, FGD group 4 P5, FGD group 8 P5, FGD group 9 P1, FGD group 10 P5 and FGD group 10 P7). “I don’t think all women are aware of cervical cancer risk factors. *We ask women about risk factors when they come for screening during counseling just before providing the screening service, but most of them don’t know any cervical cancer risk* factors.” (Almost all HCPs and family health team leaders)*

But some of focus group discussion participants tried to identify multiple risk factors such as unprotected sexual acts, poor personal hygiene, early marriage and sexual initiation, and Human Papilloma Virus (HPV) infection. They said:

*“Early marriage and sexual initiation, having sex with multiple partners (unprotected sex) can be risk factors for cervical cancer. Unprotected sex exposes to HPV and HIV infection, which can expose women to cervical cancer.” (FGD group 1 P3, FGD group 3 P4, FGD group 4 P3, and FGD group 4 P4)*

Some of the focus group discussion participants have misconceptions or beliefs about the risk factors of cervical cancer. Some women perceive or believe that a woman can be exposed to cervical cancer due to poor personal hygiene and if she urinates in sunny and hot areas.

*“Cervical cancer can occur from poor personal hygiene, and **urinating in sunny and hot areas**.” (FGD group 7 P2, FGD group 10 P10, FGD group 11 P3, and FGD group 11 P1)*

##### 3.2.2.2 Knowledge of sign and symptom

Knowledge of the signs and symptoms of cervical cancer is also a factor that can influence cervical cancer screening uptake. The most common symptom of cervical cancer identified by women was *foul-smelling vaginal discharge*. Other symptoms, such as pain during sex, bleeding, and vaginal itching, were also mentioned. Some of the focus group discussants and key interview participants said.

*“I am aware of cervical cancer symptoms, such as bad odor vaginal discharge, and itching of the vagina. Most women can identify these symptoms as cervical cancer symptoms” (FGD group 1 P7, FGD group 4 P7, FGD group 11 P5, and FGD group 11 P6).*

*Most of the women didn’t know about cervical cancer symptoms, but some of them explained symptoms like vaginal discharge, itching, and unusual frequent menstrual periods as symptoms of cervical cancer during counseling (HCP, M32-year-old Family Health Team Leader, and F32 and F30 years old Screeners).*

##### 3.2.2.3 Knowledge of prevention and control

Most focus group discussants were unaware of cervical cancer prevention methods. All key informant participants indicated that most women lacked knowledge about cervical cancer prevention methods. However, some focus group discussion participants believed that early cervical cancer screening and avoiding risk factors were effective prevention methods, and they stated:

*“Early screening and diagnosis can prevent the advancement of the disease. Additionally, cervical cancer can be prevented by maintaining proper personal genital hygiene and sanitation” (FGD group 6P7, FGD group 7 P5, and FGD group 8 P3).*

*“Cervical cancer can be prevented mainly by delaying sexual initiation during young adolescent age, avoiding early marriage, and avoiding sexual relations with men who have had multiple partners (unprotected sex), which can cause infection to their wives and lead to cervical cancer” (FGD group 3 P6).*

Focus group discussion and key informant interview participants were asked about the types of screening methods that women are aware of. Both the Focus group discussion and key informant interview participants mentioned that most women do not know about the available types of screening methods at their district health centers. Some of them said,

*“I know the screening service is available at the health center, but neither I nor the other women knew about the types of screening methods offered at the nearby health facility” (FGD group 2, P8; FGD group 8, P1; FGD group 11, P5; and FGD group 11, P6).*

*Most of the women did not know about the type of cervical cancer screening method provided in our health center. They were unaware of what kind of test it is and when it would be done (HCPs, M36-year-old FHT leaders, and F25, F28, and F31 year old screeners).*

#### 3.2.3 Theme 3: socio-cultural barriers for cervical cancer screening up taking

Under this theme, there are subthemes such as individual barriers (psychosocial barriers, spousal disapproval or resistance, and preference for the gender of the healthcare provider) and community-level barriers (fear of cancer and perception, culture, and absence of open discussion).

##### 3.2.3.1 Individual level socio-cultural barriers

###### 3.2.3.1.1 Psycho-social barriers

Psycho-social barriers, such as feeling healthy, having no symptoms, fear of divorce, fear of stigma and discrimination, fear of not being cured, fear of the test, and the cost of treatment, were raised during discussion as the main causes for not taking up screening. Focus group discussion participants were asked about their main reason for not up taking cervical cancer screening and replied that:

*Most of women do not want to up take screening since they feel healthy, do not have any symptom or do not feel any pain. Again they do not want to show their private body to other person to undergo screening (FGD group 1 P, FGD group 3 P6 FGD group 3 P1, and FGD group 8 P1).*

*I and other women fear that divorce could happen if we test positive. We worry that if our husband finds out we had cervical cancer, our marriage will fall apart. (FGD group 10 P6, FGD group 1 P3 and FGD group 3 P1),*

*Fear of discrimination and stigma is also another reason for not up taking cervical cancer screening among most women. Women think that they may discriminated by family or community if they are tested positive (FGD group 4P, FGD group 10P1and FGD group 5 P9)*

*I and most women also believe that the cost of treatment is high and we cannot afford services provided outside of government health institutions. Additionally, we worry that if we are diagnosed with cervical cancer, we will not be cured (FGD group 1 P6,and FGD group 2p2).*

*Worrying about the disease will accelerate and worsen the disease progression, and bringing death closer. Therefore, many women say, “Why didn’t I just sit down and rest, knowing that death is imminent and scary?” (FGD group 9 P5, FGD group 10 P3 and FGD group 10 P9)*

Key informant interview participants raised several issues that prevent women from utilizing screening. Factors such as fear of pain from the procedure, fear of test results, rumors about not being able to have sex after screening, infertility concerns, and feelings of shame were cited as reasons for not participating in screening among target women. Some of the KII participants said,

*“The women don’t want to be screened for various reasons, such as fear of not being cured, thinking the screening material is large and causes harm, and some women heard rumors that something will be cut and taken for screening, which causes pain. Another reason is fear of not being able to have sex after screening, and fear of not being able to give birth to a child after screening.” (30, 34, and 28 years old screening service providers, and 33, 43, and 36 years old family health team leaders)*

*“Some women get off the examination bed upon seeing the screening materials (speculum) and apologize before the speculum is inserted, refusing the screening. They express that this material hurts them; so, they do not want to be screened. Finally, they leave to home without up taking the screening.” (HCP, F33 years old cervical cancer screener)*

Another psychosocial reason revealed by many women during discussion is feeling shy and ashamed, commonly expressed by women for not up taking screening. This factor can impose a strong internal force on women not to uptake screening. Many focus group discussants and key informants reported that women feel shame in showing their private bodies to others to uptake screening, as it is considered taboo. The participants said,

*“Cervical cancer screening is something that can cause shame. When we discuss it during some gathering, many women say how can they open and show their private body to others without experiencing any symptoms? When I gave birth, I had to expose my body, as failing to do so could put my life in danger.” (FGD group 7 P5, FGD group 6 P7, FGD group 8 P8, FGD group 1 P1, and FGD group 4P7)*

*“It is shameful for women to show their private bodies to others. This may be due to our culture. Additionally, the women often say, ‘I don’t have any symptoms, so why should I need to be screened?’ Another reason may be fear of discrimination and stigma if they are diagnosed positive.” (HCP, F31, F42, and F34-year-old CCS service providers and M32 and F38 years old FHT leaders)*

###### 3.2.3.1.2 Spousal disapproval or resistance

Spousal disapproval or resistance was raised during discussions as a reason for not undergoing screening among the target women. Many participants in the Key Informant Interviews also mentioned the issue of resistance or disapproval from women’s spousal partners as a factor in their decision against screening. One of the key informant interview participants said:

*“There is pressure on women from their husbands not to be screened. After counseling, some women told me, ‘Let me talk to my husband and come back.’ While some women express they understand and want to undergo screening, but they feel the need to obtain permission from their husbands. One woman I know came for screening, and I counseled her to undergo screening. After counseling, the woman told me she would go home to ask her husband`s permission. I allowed her to go home and get permission from her husband. When I called her a few days later, she told me her husband refused. This scenario illustrates how husbands can prevent their wives from getting screened. This may be a major factor for low uptake of cervical cancer screening.” (HCP, F30-year-old screening service provider)*

During the Focus group discussions, participants were asked if their partners allowed or did not allow their wives to undergo cervical cancer screening. Some of the participants expressed the situation as:

*“Many women do not get screened due to fear of their husbands. If she goes and gets screened, the husband may ask her, ‘Where did you bring the disease from?’ He suspects that she cheated on him, and this could create problems in their marriage, possibly leading to divorce.” (FGD Group 2 P7)*

*“I don’t think partners allow their wives to undergo cervical cancer screening. Most men refuse their partner’s request for screening because they lack information about cervical cancer. Men may feel disappointed, worrying that their wives had cheated with another man, who resulted in her contracting cervical cancer, and this could ultimately lead to divorce.” (FGD Group 1 P3)*

###### 3.2.3.1.3 Preference of gender of HCP and religious matters

Another reason raised during discussion by women for not opting for cervical cancer screening was their preference for the gender of the screening service provider and religious matters. Many participants in the discussion reported that women do not want to be screened by male screening service providers. In particular, Muslim women do not want to be screened by male screeners. One of the community health leaders who was working on cervical cancer screening promotion and participated in the discussion said,

*“I advised and encouraged a number of women found in my Ketena (local administrative area) to uptake cervical cancer screening at the local district health center. When I went to the screening site, I saw many Muslim women waiting for screening services. The women who were waiting for screening told me that they would not be screened if the screener is male. We asked the health center head about the gender of the screening service provider. The health center head informed us that the screeners are female professionals. All the women were happy to see a female screener, and finally, most of them were screened.” (FGD group 11P7)*

*“My sister, one of the discussants, spoke about Muslim women in a simple way, stating that they will be screened by male screeners. But it is not as easy as she said. It is **‘HARAM’** for a Muslim woman to be touched by a man other than her husband. The Muslim women who came with me for screening told me that they would not be screened if a screener is male and said to me ‘we have a fear of **ALLAH’.** Then, I informed the head of the health center that the women need to be screened by a female screener. Finally, a female screener was assigned, and the women were screened.” (FGD group 5 P3, FGD group 11 P8)*

Key informant interview participants experienced that most women strongly prefer a female screener because they do not want to show their private body to a male screener. Most women become very happy and are more likely to undergo screening if they are screened by a female screener. Some of the key informant interview participants said:

*“Most women prefer a female screener. One of the women came and saw me in the screening room while I was providing screening services to other women. She said **‘God’** helped me and she became happy to see a female screener, saying, **‘You are female**; now I will be screened.’ When there is a male screener, most women say, ‘I don’t need to be screened.’ If the screeners are female professionals, women feel more comfortable and will be screened” (HCPs, F28, F31, and F36years old screeners)*

###### 3.2.3.1.4 Fear of infertility and perception of hereditary factors

Women have concerns about the potential harm of screening procedures and infertility associated with screening. Additionally, there is a common belief among women that cervical cancer is a hereditary disease. Thus, if there are no cases of cervical cancer in their families, they perceive that they may not have a chance to contract cervical cancer and screening is unnecessary. These views were expressed by some participants during the discussion.

*During cervical cancer screening, a material is inserted into the uterus, and some women believe the screening material can cause damage to their uterus, leading to infertility (FGD groups 8 P8, 9 P5, and 11 P4).*

*Some women believe that cervical cancer is commonly caused by hereditary factors. They say that since no woman in their family who had had cervical cancer, they do not need to undergo screening (FGD groups 8 P8, 9 P5, and 11 P4).*

##### 3.2.3.2 Community level barriers

Under this sub-thematic area, sub-themes such as fear of cancer, culture and norms, religion, and the lack of community discussions about cervical cancer are explored. Community perception regarding cervical cancer is a strong factor that may hinder women from the uptake of CCS. This perception may arise from a lack of awareness and knowledge about cervical cancer within the community.

###### 3.2.3.2.1 Community cancer perception

The community perceives that cancer is a deadly disease and believes that cervical cancer is caused by cheating or a curse from evil-doing. This perception leads to divorce, stigma, and discrimination if the woman is screened and found to be positive. Most women do not want to lose their family because of this reason. One of the key informants said,

*“The community has a fear of cancer, thinking it is a deadly disease. If a woman contracts cervical cancer, her husband may divorce her; he will not take care of her because he assumes that she cheated on him and/or she is cursed. Therefore, the women do not come to the health center to screen for cervical cancer.” (36-year-old family health team leader).*

*“Society’s attitude towards cervical cancer is negative. The community believes that cervical cancer will occur due to a curse or cheating by the woman” (HCP, F28-year-old screener).*

###### 3.2.3.2.2 Culture and norms

Community culture can influence the health-seeking behavior of the individuals, particularly regarding reproductive healthcare services like cervical cancer screening. Most discussants believe that the community views exposing private body parts to others as shameful, or forbidden, even for health care providers, when the woman has no disease or symptoms. Some of the study participants said:

*“It is considered shameful for women in our culture to show their private bodies to another person, except for their husbands. Therefore, women feel ashamed to show their private bodies to healthcare providers. This could be a reason why women are not being screened”. (FGD group 6 P7).*

###### 3.2.3.2.3 Absence of open community discussion

Community open discussion has been used to address issues such as discrimination, disclosure, early screening, and acceptance of chronic HIV care and support worldwide. This experience is vital for the elimination of cervical cancer. During the focus group discussion, participants were asked if there had been any discussion about cervical cancer in their families and communities. Many of them shared their experiences. Some of the discussants stated:

*“I never discussed cervical cancer with my husband. He does not know anything about cervical cancer, and he refuses to talk about it when I try to discuss it with him. Whenever the topic of cervical cancer comes up on the radio or TV, he orders us to switch to another program. This is the reality in our family not to discuss. Husbands do not want to hear about cervical cancer. There is a lack of awareness and a belief that the disease is scary and terrifying.” (FGD group 6 P9)*

*The community avoids openly discussing cervical cancer and prefers not to address the issue of cervical cancer. When we encourage men to have their partners screened, they deny it as something that the government is promoting. They do not believe that the disease exists in our country. As a result, they prevent their wives from undergoing screening. Men exert pressure on women to avoid screening. (FGD group 2 P8, FGD group 8 P4, and FGD group 9 P6)*

#### 3.2.4 Theme 4: expectations of study participants

Most study participants believe that cervical cancer is a major cause of morbidity and mortality among women nowadays. Prevention and control of the disease requires collaborative work among the government, the community, and families. The expectations and support needed by women were also discussed with the study participants, and some women said that

*A higher contribution is expected from the government to create open community discussion and awareness creation among the community, similar to what had been done for HIV/AIDS. Health education should be provided through the media and at community gatherings, aiming to raise awareness. Every family and husband should listen to the media and health extension teaching in order to be aware and support each other. Husbands should accompany and screen their wives and hear the results of the screening. If there is a problem, he should be involved and take care of his wife. (FGD group 1 P6, FGD group 4P2, FGD group 5 P6, FGD group 10 P4, FGD group 8 P5)*

## 4 Discussion

The result of this study showed most women lack knowledge about the risk factors, symptoms, and prevention of cervical cancer. The findings of this study is supported by studies conducted in various areas of Ethiopia, including Addis Ababa, East Gojam and Addis Ababa, Kambata Tambaro, and Hadiya Zone and Arba Minch (18, 22–24), Kenya (17), Lagos, Nigeria (25), China (26) and Minnesota among Somali immigrants (15). The similarity may be due to the fact that hearing alone may not help women understand cervical cancer in detail. Poor knowledge about cervical cancer and screening can significantly hinder women’s uptake of screening. It is important to look for strategies to increase knowledge about cervical cancer and screening by providing comprehensive, culturally sensitive educational campaigns, improved access to healthcare services, and efforts to enhance health literacy related to cervical cancer screening among women.

The results of this study showed that feeling healthy and having a low-risk perception were the reasons for not taking up screening. This is supported by studies conducted in southern Ethiopia (23, 24), Ecuador (27), and Minnesota among Somali immigrants (15). This may be due to a lack of knowledge about risk factors and symptoms. Feeling healthy and perceiving being at low risk leads to a false sense of security and a belief in being free from diseases. This results in a late and advanced-stage diagnosis. Therefore, targeted health education is crucial to improving knowledge of risk factors and symptoms so that women understand they are at risk of developing the disease. Feeling at risk motivates women to screen for cervical cancer.

Another reason mentioned by the women for not taking cervical cancer screening was fear of screening materials, fear of pain, and discomfort. The results of this study are similar to the studies conducted in Addis Ababa and Arba Minch (22, 24), Ecuador (27), Kenya (17), Ghana (16), Cameroon (28) and Minnesota among Somali immigrants (15). The similarity may be due to misconceptions and the belief that the material can hurt them, causing problems in their reproductive organs, which leads to a loss of sexual activity and, ultimately divorce. It is essential to implement strategies such as education and counseling so that the women understand that screening materials do not cause any significant pain, do not hurt them, or do not lead to loss of sexual activity.

The result of this study found that women’s feelings of shame and embarrassment from exposing their private bodies to others during cervical cancer screening are the cause of low screening uptake. This finding is supported by study conducted in Addis Ababa and Arba Minch, southern Ethiopia (22–24), Ecuador (27), Iran (29), and China. The similarities can be attributed to the influence of community culture and norms that discourage women from exposing their private bodies to others. Religiously, it is illegal to show private bodies (reproductive organs) to others, especially among Muslim believers; this may be a key reason for not up taking cervical cancer screening. Behavioral communication and change activities are necessary to shift social norms and reduce feelings of shame associated with the uptake of cervical cancer screening.

Another result of this study showed that spousal approval and support have a greater influence on cervical cancer screening uptake and are considered as one of the main reasons for not taking up screening. The result of this study is supported by studies conducted in Addis Ababa and East Gojam, Ethiopia (18, 22), African countries such as Kenya, Nigeria, and Cameroon (17, 25, 28, 30) and Ecuador (27). The similarity may be attributed to the cultural belief that women need permission from male partners to access healthcare services, especially preventive healthcare services. If women undergo cervical cancer screening without their husband’s permission, the husband may perceive it as infidelity, and he may decide to divorce her. Creating awareness among male partners and community engagement in awareness creation helps to improve spousal support.

The gender of the screening service provider was raised as the reason for not up taking of cervical cancer screening. The majority of women prefer a female cervical cancer screening service provider. The findings of this study are supported by studies conducted in Addis Ababa (22), Lagos, Nigeria (25), rural Nigeria (30), Ecuador (27), and Singapore (31). The similarity may be due to the fact that women may feel ashamed to show their private bodies to male screening service providers. For Muslim women, it is not allowed to be touched on by another male except her husband. The gender of the screening service provider can affect uptake of screening, particularly in communities where gender norms and preferences play a significant role in healthcare-seeking behavior. Client-centered approaches and culturally sensitive care in healthcare systems (provided by female healthcare providers) can improve screening uptake.

The results of this study identified fear of cancer as a reason for not up taking cervical cancer screening. The result of this study was supported by the study conducted in Addis Ababa (22) and Wolaita (32). This may be due to a lack of awareness and knowledge about CCa and CCS. The community is unaware that early-diagnosed and treated cervical cancer can leads to cure and prevention. Raising awareness about the benefits of early cervical cancer screening is a crucial to increase the uptake of CCs and preventing further damage and loss of life due to cervical cancer.

The fear of stigma and discrimination associated with cervical cancer screening was raised as a reason for not undergoing screening. The results of the study are supported by studies conducted in Addis Ababa, East Gojam, Southern Ethiopia, and Arba Minch, Southern Ethiopia (18, 22–24), Cameroon (28), Ecuador (27), and two cities in China (26). The similarity may be due to the observation that women suffering from cervical cancer were stigmatized and discriminated against by the community and eventually died. Stigma and discrimination due to cervical cancer can be manifested in several ways. Women diagnosed with cervical cancer may face social isolation, blame, and exclusion from their communities. This societal attitude often stems from misconceptions, such as associating cervical cancer with promiscuity or moral judgment. Such stigmatization not only affects the psychological well-being of affected individuals but also acts as a deterrent for others considering cervical cancer screening. Implementing comprehensive community awareness programs, engaging local community leaders, religious leaders, and influential figures to change societal attitudes, ensuring that cervical cancer screening services are accessible and affordable, establishing peer support groups and counseling services for women diagnosed with cervical cancer, and advocating for policies that protect individuals from discrimination based on health status are crucial.

This study revealed a lack of open community discussion as a reason for not up taking cervical cancer screening. The result of this study is supported by the study conducted in Iran (29). In Ethiopia, discussions about sexual and reproductive health are often considered taboo (33). Because of the lack of open community discussion about cervical cancer, male partners lack awareness and disapprove of the screening up take of their wives. The cultural taboo of discussing reproductive organs results in a lack of open community dialogue about cervical cancer, which in turn impacts women’s access to information, support from their male partners, and ultimately their decision to undergo screening. Open discussions increase awareness among both men and women about the importance of cervical cancer screening help break down the stigma associated with reproductive health issues, including cervical cancer. Therefore, creating open community dialogue, engaging religious leaders and community elders to endorse and promote open discussions about cervical cancer, and establishing peer education groups are important to increase the uptake of cervical cancer screening.

The study participants expect that the government should facilitate open community discussions to increase awareness and knowledge among the entire community and provide health education for the male community, which could help increase spousal support that influences women’s uptake of screening.

### 4.1 Strength and weakness

The data were collected from all 11 sub-cities. Therefore, it can be generalized to the entire female population in Addis Ababa city. However, there is a lack of similar literature to compare the results of this study with other studies in a similar context.

## 5 Conclusion and recommendations

In this study, knowledge of cervical cancer and screening was found to be poor. Individual-level socio-cultural factors, such as feeling healthy, having a low perception of risk, fear of tests and procedures, fear of divorce, stigma and discrimination, fear of not being cured, preference for the gender of healthcare providers, and spousal disapproval or resistance, were identified as the main reasons for the low uptake of cervical cancer screening. The community perceives cervical cancer as a deadly disease. Misconceptions, such as screening materials causing pain and infertility, and the lack of open discussion, contribute to the low uptake of cervical cancer.

To address the barriers highlighted in the study and increase the uptake of cervical cancer screening, focused interventions and strategies are critical. This includes community-wide educational programs to combat misinformation, providing gender-sensitive healthcare services, establishing family or peer support for women to undergo cervical cancer screening or treatment, and offering counseling services to address concerns about the screening process, diagnosis, and treatment outcomes.

## Data Availability

The datasets used and/or analyzed during the current study are available from the corresponding author upon reasonable request.
