# Correction: Reasons for not undergoing cervical cancer screening: perspectives from women and health care providers in Addis Ababa: a qualitative study

**DOI:** 10.3389/fonc.2025.1650395

**Published:** 2025-07-03

**Authors:** Ebrahim Mohammed, Mirgissa Kaba, Girma Taye, Mathewos Assefa, Ahmedin Jemal, Adamu Addissie

**Affiliations:** ^1^ Department of Reproductive, Family and Population Health, School of Public Health, Addis Ababa University, Addis Ababa, Ethiopia; ^2^ Department of Preventive Medicine, School of Public Health, Addis Ababa University, Addis Ababa, Ethiopia; ^3^ Department of Epidemiology and Biostatistics, School of Public Health, Addis Ababa University, College of Health Sciences, Addis Ababa, Ethiopia; ^4^ Department of Oncology, College of Health Sciences, School of Medicine, Addis Ababa University, Addis Ababa, Ethiopia; ^5^ Surveillance & Health Equity Science, American Cancer Society, Atlanta, GA, United States

**Keywords:** cervical cancer, cervical cancer screening, perspectives on cervical cancer, women, healthcare providers

In the published there was a mistake on authors’ affiliation. The authors’ affiliation was written as

“^1^Department of Public Health, Adama Hospital Medical College, Adama, Ethiopia, ^2^Department of Preventive Medicine, School of Public Health, Addis Ababa University, Addis Ababa, Ethiopia, ^3^Department of Epidemiology and Biostatistics, School of Public Health, Addis Ababa University, College of Health Sciences, Addis Ababa, Ethiopia, ^4^Department of Oncology, College of Health Sciences, School of Medicine, Addis Ababa University, Addis Ababa, Ethiopia, ^5^Surveillance & Health Equity Science, American Cancer Society, Atlanta, GA, United States”

It should be corrected as

“^1^Department of Reproductive, Family and Population Health, School of Public Health, Addis Ababa University, Addis Ababa, Ethiopia, ^2^Department of Preventive Medicine, School of Public Health, Addis Ababa University, Addis Ababa, Ethiopia, ^3^Department of Epidemiology and Biostatistics, School of Public Health, Addis Ababa University, College of Health Sciences, Addis Ababa, Ethiopia, ^4^Department of Oncology, College of Health Sciences, School of Medicine, Addis Ababa University, Addis Ababa, Ethiopia, ^5^Surveillance & Health Equity Science, American Cancer Society, Atlanta, GA, United States”

The original version of this article has been updated.

