# Supplementary material for: Reasons for not undergoing cervical cancer screening: perspectives from women and health care providers in Addis Ababa: a qualitative study
Source: Front Oncol. 2025 Apr 2;15:1456804. doi: 10.3389/fonc.2025.1456804 (PMC11999823; doi:10.3389/fonc.2025.1456804)
Supplement: Supplementary file 1 [file DataSheet1.docx]

**FGDs Interview Guide**

 Part I: Socio-cultural Variables

1. Please introduce yourself:

a. Age: ________________

b. Educational Status: ________________

c. Occupation: ________________

d. Position in the Community: ________________

 **Part II:** **Awareness Related to Cervical Cancer and Screening**

1. Have you heard about cervical cancer before? Where do you get information about cervical cancer? Where would you like to get this information? Who told you about cervical cancer?
2. What do you think is the cause of cervical cancer?

Probes: What, in general, do you think causes cervical cancer? What about HPV and cervical cancer?

1. What are the risks of contracting cervical cancer?

Probe: What puts people at risk for cervical cancer? What risks do you think are most common in your community?

1. What are the signs and symptoms of cervical cancer? Please describe them.
2. What helps to protect people from cervical cancer?
3. Have you heard about cervical cancer screening?
4. What are some methods you know to screen for cervical cancer? Probe: Which screening methods are commonly used by community members?
5. What type of practice is there in the community related to cervical cancer screening? Probe: Is cancer is worry for your community? Does the community discuss cancer openly? What about cancer screening? Does the community think pre-cancerous screening is important, especially cervical cancer screening?

**Part III:** Attitudes, Behaviors

1. Most women don’t want to undergo cervical cancer screening. What is the reason for not undergoing the screening? Probe: Please describe the reasons in detail.
2. What type of support do you think is important for women to undergo cervical cancer screening? Example: health education, human resource availability, availability of services.
3. Do you think cervical cancer screening could prevent cervical cancer (reduce the chance of contracting cervical cancer)?
4. What is the community perception toward cervical cancer precancerous screening?
5. Do you think cervical cancer screening helps to prevent cervical cancer?

**Part IV :** Socio-Cultural related questions

1. How do you feel if you are recommended to have pelvic and reproductive organ exams?

Probes: Would you skip cervical cancer screening because it involves a pelvic exam? Do you know a woman in your community who does not participate in CCS due to the pelvic examination?

1. Do you feel comfortable seeing a health professional examining pelvic or other reproductive organ issues?
2. What gender of health care professionals do you prefer? Can you tell me the reason?
3. What support should the government, your partners, family, friends, and community provide to encourage you to participate in CCS?
4. Do you have any other comments?

Thank you!!
