# Supplementary material for: Reasons for not undergoing cervical cancer screening: perspectives from women and health care providers in Addis Ababa: a qualitative study
Source: Front Oncol. 2025 Apr 2;15:1456804. doi: 10.3389/fonc.2025.1456804 (PMC11999823; doi:10.3389/fonc.2025.1456804)
Supplement: Supplementary file 2 [file DataSheet2.docx]

**KIIs Interview Guide**

Part I: Socio-cultural variables

1. Please, introduce yourself,
   1. Age __________
   2. Educational status ____________
   3. Profession___________
   4. Role _______________

**Part II:** **Awareness related to cervical cancer and screening**

1. Have the women heard about cervical cancer and screening before? Where do you get information about cervical cancer? Where would you like to get this information? Who told you about cervical cancer?
2. Do you think women know the cause of cervical cancer?
3. Do you think women know about the risks of contracting cervical cancer? Do they know?
4. Do you think women know about the symptoms of cervical cancer? Please describe them.
5. Do you think women have heard about cervical cancer screening?

**Part III:- Cervical cancer screening uptake**

1. Most women don’t want to uptake cervical cancer screening. What is the reason for not up taking the screening? Probe: Please describe the reasons in detail.
2. Do you think the provision of cervical cancer screening by a male or female screener creates a difference in the acceptance of cervical cancer screening? Could you describe the difference?

**Part IV: Community perception**

1. What do you think the perception of the community is about cervical cancer and screening?
2. What should the husband, family, community, and government do so that women can take up cervical cancer screening?
3. Have you faced a shortage of screening materials?
4. Do you have any additional ideas?

Thank you!!
